# Tor Signaling Regulates Transcription of Amino Acid Permeases through a GATA Transcription Factor Gaf1 in Fission Yeast

**DOI:** 10.1371/journal.pone.0144677

**Published:** 2015-12-21

**Authors:** Yan Ma, Ning Ma, Qingbin Liu, Yao Qi, Ri-ichiroh Manabe, Tomoyuki Furuyashiki

**Affiliations:** 1 Division of Pharmacology, Kobe University Graduate School of Medicine, Kobe, Japan; 2 Division of Genomic Technologies, RIKEN Center for Life Science Technologies, Yokohama, Japan; Newcastle University, UNITED KINGDOM

## Abstract

In the fission yeast, two Tor isoforms, Tor1 and Tor2, oppositely regulate gene expression of amino acid permeases. To elucidate the transcriptional machinery for these regulations, here we have employed the cap analysis of gene expression (CAGE), a method of analyzing expression profiles and identifying transcriptional start sites (TSSs). The loss of Tor1 decreased, and Tor2 inhibition by its temperature sensitive mutation increased, mRNA expression of *isp5* ^*+*^, *per1* ^*+*^, *put4* ^*+*^ and *SPBPB2B2*.*01*. In contrast, the loss of Tor1 increased, and Tor2 inhibition decreased, the expression of *cat1* ^*+*^. These changes were confirmed by semi-quantitative RT-PCR. These opposite effects by the loss of Tor1 and Tor2 inhibition appeared to occur evenly across multiple TSSs for the respective genes. The motif discovery analysis based on the CAGE results identified the GATA motifs as a potential *cis*-regulatory element for Tor-mediated regulation. In the luciferase reporter assay, the loss of Tor1 reduced, and Tor2 inhibition and nitrogen depletion increased, the activity of *isp5* ^+^ promoter as well as that of a GATAAG reporter. One of the GATAAG motifs in *isp5* ^+^ promoter was critical for its transcriptional activity, and a GATA transcription factor Gaf1 was critical for the activities of *isp5* ^+^ promoter and the GATAAG reporter. Furthermore, Tor2 inhibition and nitrogen depletion induced nuclear localization of Gaf1 from the cytosol and its dephosphorylation. These results suggest that Tor2 inhibition, which is known to be induced by nitrogen depletion, promotes nuclear localization of Gaf1, thereby inducing *isp5* ^+^ transcription through Gaf1 binding to the GATAAG motif in its promoter. Since Gaf1 was also critical for transcription of *per1* ^+^ and *put4* ^+^, Tor-Gaf1 signaling may coordinate transcription of multiple amino acid permeases according to nutrient availability.

## Introduction

The target of rapamycin (TOR) is a structurally and functionally conserved protein kinase that plays a key role in integrating nutrient and energy signaling to promote cell proliferation and growth [1–3]. TOR proteins are found at the core of two evolutionarily conserved complexes, known as TORC1 and TORC2. TORC1 regulates cell growth in response to nutrient availability, growth factors and energy status by inhibiting catabolic processes and promoting anabolic processes at multiple levels including protein translation, ribosome biogenesis, gene transcription, protein degradation and autophagy [4, 5]. In fission yeast *Schizosaccharomyces pombe*, Tor2, a core kinase in TORC1, links nitrogen signals to cell proliferation [6]. Nitrogen depletion suppresses Tor2 activity, and nitrogen starvation-responsive genes are largely controlled by Tor2 [7]. Several amino acid permeases are included in these genes. Amino acid permeases are a family of proteins that mediate amino acid flux with distinct substrate specificity and subcellular localization [8]. Tor2 regulates transcription of respective amino acid permeases in distinct manners, either positively or negatively [9, 10]. In addition, Tor1, a core kinase in TORC2, is shown to be involved in transcription of several amino acid permeases in a manner distinct from Tor2 [10]. However, the underlying mechanism remains to be determined. To address this issue, it is critical to identify *cis*-regulatory elements and transcription factors which mediate Tor signaling for transcriptional regulation of amino acid permeases.

In the last few years, the cap analysis of gene expression (CAGE) has been developed to study the transcriptional regulation on a genome-wide scale [11–13]. This analysis allows high-throughput identification of sequence tags corresponding to 5’ ends of mRNA at the cap sites, thereby facilitating the prediction of core promoters [13, 14]. Using this method, here we have found that Tor1 and Tor2 oppositely regulate transcription of several amino acid permease genes, namely *isp5* ^*+*^, *per1* ^*+*^, *put4* ^*+*^, *SPBPB2B2*.*01*, and *cat1* ^*+*^, and that these opposite regulations are evenly distributed across multiple TSSs of the respective genes. The motif discovery analysis based on the CAGE results revealed the GATA motif as a potential *cis*-regulatory element for Tor-mediated transcription, and multiple lines of evidence suggest a critical role of a GATA transcription factor Gaf1 in Tor signaling for regulating transcription of amino acid permeases.

## Materials and Methods

### Yeast strains, Growth Media, Drugs and General Methods

The *Schizosaccharomyces pombe* strains used in this study are listed in Table 1. The media (EMM and nitrogen-depleted EMM), denotation and genetic methods have been described previously [15, 16]. Gene disruptions are indicated by the gene symbol preceded by Δ (for example, Δ*tor1*). Proteins are denoted by Roman letters with only the first letter capitalized (for example, Tor2). Drugs were obtained from the following sources: DMSO (Nacalai), rapamycin (Toronto Research Chemicals) and Torin-1 (Tocris). Database searches were performed using Pombe community database PomBase (http://www.pombase.org).

**Table 1:** *Schizosaccharomyces pombe* Strains used in this Study.

Strain	Genotype	Reference
HM123	*h* ^*-*^ *leu1-32*	Our stock
KP456	*h* ^*-*^ *leu1-32 ura4-D18*	Our stock
KP928	*h* ^*+*^ *his2 leu1-32 ura4-D18*	Our stock
KP207	*h* ^*+*^ *his2 leu1-32*	Our stock
KP5482	*h* ^*-*^ *leu1-32 tor2-287*	[7]
KP3222	*h* ^*-*^ *leu1-32 ura4-D18 tor1*::*ura4* ^*+*^	This study
KP5080	*h* ^*-*^	[9]
KP5734	*h* ^*+*^ *tor2-287*	[9]
KP5733	*h* ^?^ *ura4-D18 tor1*::*ura4* ^*+*^	This study
KP5079	*h* ^*-*^ *ura4-D18*	This study
KP6311	*h* ^*-*^ *leu1-32 ura4-D18 tor2-287 tor1*::*ura4* ^*+*^	This study
KP6488	*h* ^*-*^ *ura4-D18 Gaf1-YFP*::*ura4* ^*+*^	This study
KP6489	*h* ^*+*^ *ura4-D18 Gaf1-YFP*::*ura4* ^*+*^ *tor2-287*	This study
KP6502	*h* ^?^ *ura4-D18 Gaf1-YFP*::*ura4* ^*+*^ *tor1*::*ura4* ^*+*^	This study
KP90964	*h* ^*+*^ *leu1-32 ade6 ura4-D18 gaf1*::*KanMX*_*4*_	[17]
KP5835	*h* ^*+*^ *leu1-32 gaf1*::*KanMX*_*4*_	This study
KP5837	*h* ^*-*^ *gaf1*::*KanMX*_*4*_	This study
KP6680	*h* ^*-*^ *ura4 gaf1*::*KanMX*_*4*_	This study

### Generation of Δ*tor1* Strain

The Δ*tor1* strain was generated using a one-step gene disruption by homologous recombination [18]. The *tor1*::*ura4* ^+^ disruption was constructed as follows: The BamHI fragment containing *tor1* ^+^ was subcloned into the BamHI site of pGEM-7Zf (Promega). Then, a PstI fragment containing *ura4* ^+^ was inserted into the PstI site of the previous construct. The resulting plasmid pKB6751 was digested with BamHI, and the fragment containing the disrupted *tor1* ^+^ gene was transformed into KP456 (*h* ^*-*^ *leu1-32 ura4-D18*). Stable integrants were selected on media lacking uracil, and disruption of the gene was checked by genomic Southern hybridization (data not shown).

### CAGE Library Preparation, Sequencing and Data Analysis

The prototrophic wild-type, *tor2-287* mutant and Δ*tor1* cells were cultured in EMM media to log-phase. After the cells were collected, the total RNA was prepared using the RNeasy Mini Kit (Qiagen) with on-column deoxyribonuclease digestion (RNase-free DNase Set, Qiagen). The no-amplification non-tagging CAGE library was prepared as described previously [19] using 5 μg total RNA with RIN value of more than 9. The library was subjected to 50-base single read sequencing on a HiSeq 2000 sequencer. The sequencing generated 10 to 20 million reads from each sample. The reads with low-quality and ambiguous bases and corresponding to ribosomal RNA were removed using fastX tool kit [http://hannonlab.cshl.edu/fastx_toolkit] and rRNAdust [http://fantom.gsc.riken.jp/5/sstar/Protocols:rRNAdust], respectively. The remaining reads were mapped to *S*. *pombe* reference genome using bwa [20]. CAGE read clustering, differential gene expression analysis and motif discovery were performed by RECLU ver3.1 pipeline (http://cell-innovation.nig.ac.jp/wiki2/tiki-index.php?page=P000001286), integrated into Maser (National Institute of Genetics; https://cell-innovation.nig.ac.jp/index_en.html) using default settings except with > 1.0 absolute log(2) fold-change for differential expression analysis. The distribution of TSSs at 1bp resolution is visualized by Integrative Genomics Viewer (IGV) (Broad Institute). Reproducible results were obtained from two independent samples for respective groups.

### RNA Extraction and Semi-quantitative RT-PCR

Total RNA was extracted from yeast cells using the RNeasy Mini kit (Qiagen) with on-column deoxyribonuclease digestion (RNase-Free DNase Set; Qiagen). cDNA was synthesized from the resultant total RNA using the High Capacity cDNA Reverse Transcription Kit (ABI) and subjected to semi-quantitative PCR with the SYBR Green PCR Master Mix (ABI). The primers for RT-PCR were summarized in Table 2. Signals were detected and analyzed with an Applied Biosystems 7500 Real-Time PCR System (ABI). The mRNA levels of amino acid permeases were normalized to those of *act1* according to the comparative CT method, and were statistically analyzed.

**Table 2:** Primers used for semi-quantitative RT-PCR.

mRNA	Sequence
*isp5 sense*	TCGGTGTACGAGGTTATGGT
*isp5 antisense*	GGTGGAAAAGACAGAGCAGA
*per1 sense*	ACTACTGCCGGTTTCTCCTT
*per1 antisense*	TAGCAATACGCCAAAAGACC
*put4 sense*	AGTGCCGTGATTAAAAGTGG
*put4 antisense*	TGCTAGTGGCTTTAGGGATG
*spbpb2b2*.*01 sense*	TTTGCTGGGGCAGTATATGT
*spbpb2b2*.*01 antisense*	AACCTCTTTGAGGCTCCTGT
*cat1 sense*	GTTTCGACATGGGTTCAAAG
*cat1 antisense*	AACTTGCTTAACGGCATGAG
*act1 sense*	ATCCAACCGTGAGAAGATGA
*act1 antisense*	ACCATCACCAGAGTCCAAGA

### Luciferase Reporter Assays

A Renilla luciferase reporter plasmid for the 1,038bp region from the translation initiation site of *isp5* ^+^ promoter (pKB8527) was previously constructed and reported [9]. The CAGE data in this study revealed that the actual transcriptional start site (TSS) of *isp5* ^+^ (chr1:5466753) is much closer to the translation initiation site (chr1:5466777) than that in the current PomBase data (chr1:5465377). Based on this new TSS, the 1038bp region from the translation initiation site contains 1013bp region from the TSS, and this promoter fragment was designated *isp5* ^-1013^ in this study rather than our previous nomenclature (i.e. *isp5* ^-1038^) [9]. A novel reporter plasmid for the 513-bp region from the TSS of *isp5* ^+^ promoter (*isp5* ^-513^) was generated using the following primers: sense primer (4017) 5’-AAC TGC AGC TCC GGA TTT ATA AGA GCG-3’ and antisense primer (3941) 5’-CCG CTC GAG TTT AAT TTT TTG TTT GAT GG-3’. The resultant fragment was subcloned into the PstI/XhoI-digested pKB5878 [21], a phRG(R2.2)-basic multicopy vector (Promega) that contains Renilla luciferase reporter gene. The resulting plasmid was registered as pKB8632 (*isp5* ^-513^). A reporter plasmid with the 513-bp region of *isp5* ^+^ promoter lacking the GATAAG motif (*isp5* ^-513Δ^) was constructed from pKB8632 by introducing the deletion of this motif by PCR using a sense primer (4983) 5’-GCT TGG CAA AAA TTG TTT TGT AAA TTG AAT GAA AAA ATG-3’ and an antisense primer (4982) 5’-CGA ACC GTT TTT AAC AAA ACA TTT AAC TTA CTT TTT TAC-3’. The resulting plasmid was registered as pKB9112 (*isp5* ^-513Δ^).

A Renilla reporter plasmid for the GATAAG motif containing three tandem copies of GATAAGATAAG was constructed as described previously [22], except that a *cis*-regulatory element was generated by annealing the following oligonucleotides: sense primer (4548) 5’-GGC TTT GAT AAG ATA AGA TAC ATG ATA AGA TAA GAT ACA CAT GAT AAG ATA AGA TGC AC-3’ and antisense primer (4549) 5’-TCG AGT GGA TCT TAT CTT ATC ATG TGT ATC TTA TCT TAT CAT GTA TCT TAT CTT ATC AAA GCC TGC A-3’. The resulting plasmid was registered as pKB8742 (3xGATAAG Renilla reporter).

A Renilla reporter plasmid for the CDRE (calcineurin-dependent response element) motif containing three tandem copies of AGCCTC was constructed as follows. The open reading frame of firefly luciferase of pKB5723 (3xCDRE firefly reporter) [22] was replaced by the NcoI/NotI fragment from pKB5878 [21] containing the open reading frame encoding Renilla luciferase. The resulting plasmid was registered as pKB9132 (3xCDRE Renilla reporter).

To measure the Renilla luciferase activity, the cells transformed with a reporter plasmid were added with the substrate, coelenterazine (Promega), and the bioluminescence was real-time detected as relative light units (RLU) at 27°C every minute using a microplate luminometer (AB-2350, ATTO Co.), as described previously [9]. Since the bioluminescence signals were peaked at about one hour in our conditions, we measured RLU for at least 2 hours. The peak RLU value during this period was determined and used for statistical analyses.

### Construction of the Cells Expressing Gaf1-YFP under its Native Promoter and Fluorescent Imaging Analyses of Gaf1-YFP Localization

The yeast strain expressing Gaf1-YFP under its native promoter (KP6488) was generated as follows. A plasmid containing the open reading frame of Gaf1-YFP-FLAG-His_6_ (Orfeome clone 37/H01) was purchased from RIKEN BioResource Center. This plasmid registered as pKB8931 was digested with KpnI to obtain the DNA fragment containing the open reading frame encoding Gaf1-YFP-FLAG-His_6_ and *ura4* ^+^ marker, and this DNA fragment was integrated into the chromosome at the *gaf1* ^+^ gene locus of KP5079 (*h* ^*-*^ *ura4-D18*) by homologous recombination.

Fluorescent images were acquired using a microscope (Axioskop 2 Plus; Carl Zeiss, Germany) equipped with an alpha Plan-Fluor 100x/N.A.1.45 oil objective lens (Carl Zeiss) and a SPOT 2 digital camera in combination with the Spot32 software version 2.1.2 (Diagnostic Instruments, Sterling Heights, MI). Fluorescent images were processed using Adobe Photoshop CS6 only for illustrative purposes.

### Immunoblot Analyses

A plasmid to express Gaf1-GFP under the *nmt41* promoter was generated as described below. The *gaf1* ^+^ gene was amplified by PCR with the genomic DNA of wild-type cells as a template. The sense primer was (4582) 5’-CCG CTC GAG ATG GAT CTA AAG TTT TCC-3', and the antisense primer was (4583) 5’-CCG CTC GAG GCG GCC GCC CAT AAC GCT ATA CCA ATC C-3’. The amplified product containing the *gaf1* ^+^ gene was digested with XhoI, and the resulting fragment was subcloned into the XhoI site of BlueScriptSK (+) (Stratagene). Then, a XhoI/NotI fragment containing *gaf1* ^+^ was ligated to the XhoI/NotI site of the C terminus of the GFP carrying the S65T mutation [23]. The cells were transformed with the resultant plasmid (pKB9124) and cultured on EMM with thiamine (50 μM). The transformants were grown in fresh EMM without thiamine for 20 hours to induce expression of Gaf1-GFP.

To examine Gaf1 phosphorylation, immunoblot analyses of Gaf1-GFP were performed, as previously described [24]. λ protein phosphatase assay was performed, as previously described [16] with minor modifications. Briefly, cells were resuspended in the lysis buffer (50 mM Tris-HCl (pH 7.5), 150 mM NaCl, 5 mM EDTA, 10% glycerol, 0.2% NP-40, 20 mM β-glycerophosphate, 0.1 mM Na_3_VO_4_, 10 mM p-nitrophenyl phosphate, 10 mM NaF, 1 mM dithiothreitol, 1 mM phenylmethylsulfonyl fluoride, and Halt Protease Inhibitor Cocktail, EDTA-Free (Thermo scientific)) and disrupted with microbeads. The resultant lysate was diluted by 4-fold into 1 x PMP buffer supplied by the kit with 2 mM MnCl_2_ and then incubated with λ protein phosphatase (NEB) at 30°C for 40 min without or with its inhibitor cocktails (50 mM EDTA, 10 mM Na_3_VO_4_, and 50 mM NaF). For other immunoblot analyses, the cell lysate was prepared using a 1.85M NaOH containing β-mercaptoethanol (7.5% v/v), and was neutralized with 50% trichloroacetic acid (TCA). The resultant lysates were subjected to SDS-PAGE with 10% precast polyacrylamide gels (Nacalai). Proteins were then transferred onto PVDF membrane. After blocking, the membrane was incubated with rabbit anti-GFP antibody (1:5000 dilution) [25] or mouse anti-α-tubulin antibody (1:10000 dilution, clone B5-1-2, Sigma-Aldrich). Rabbit anti-phospho-(Ser/Thr) Akt antibody (#9611, Cell Signaling Technology) was used to detect Rps6 phosphorylation as a readout for Tor2 activity, as previously reported [24]. The resultant membrane was further incubated with HRP-conjugated anti-rabbit or anti-mouse antibody (CST). Signals were detected using Clarity Western ECL Substrate (BioRad) and autoradiography films (FUJIFILM).

### Statistical Analyses

Data are shown as means ± SEM. Comparison between two groups was statistically analyzed with unpaired *t*-test. Comparison of more than two groups was statistically analyzed with one-way ANOVA followed by Tukey’s multiple comparison test to evaluate pairwise group differences. Planned comparisons were performed for selected comparison groups, if it is necessary to assess statistical significance for the difference which could not be detected with multiple comparison tests across all the comparison groups. The *P* values less than 0.05 are considered to be significant. Statistical analyses were performed with Prism 6 (GraphPad).

## Results

### Tor1 and Tor2 Oppositely Regulate mRNA Expression of Several Amino Acid Permeases

**Fig 1:**
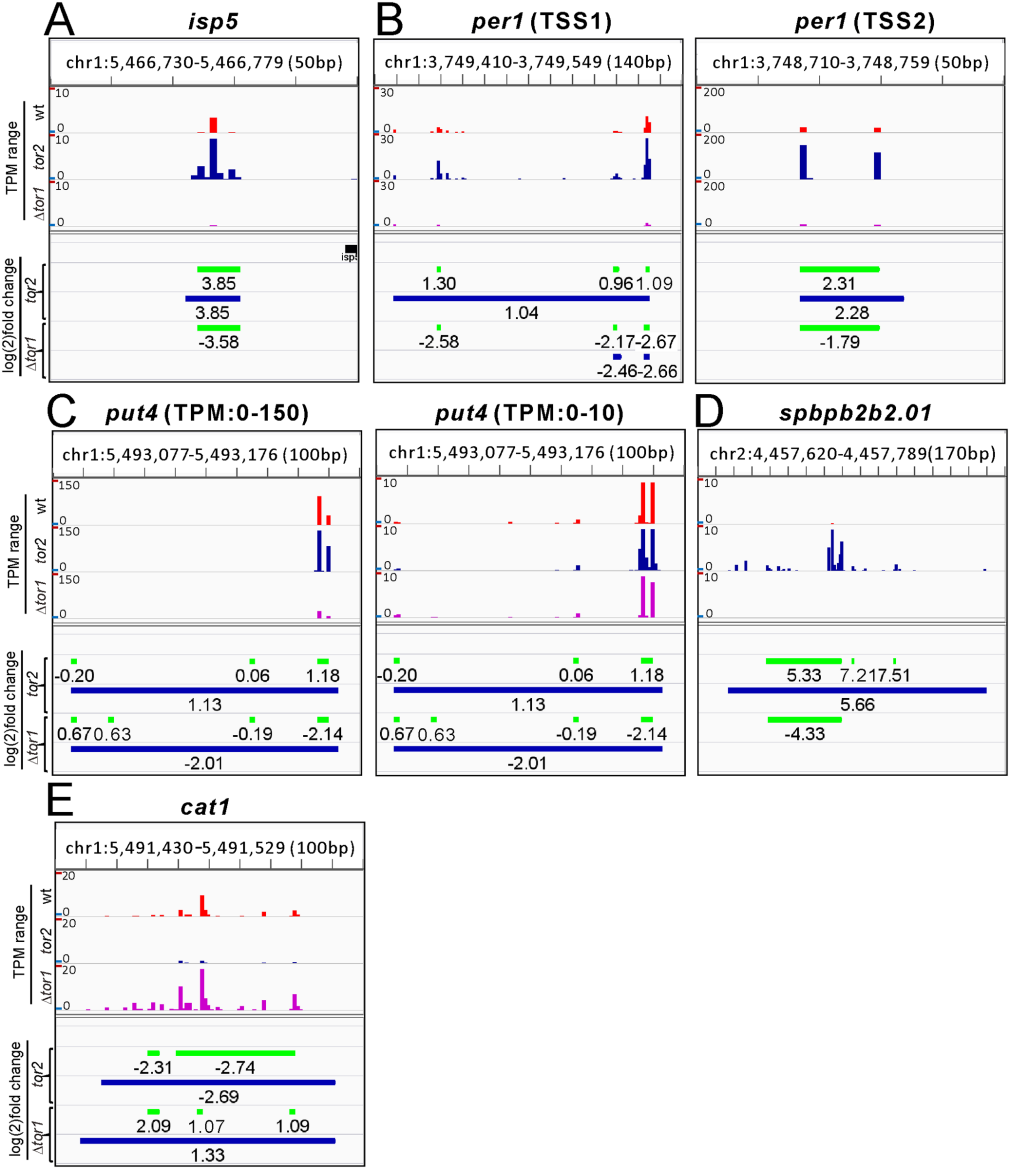
Opposite regulations by the loss of Tor1 and Tor2 inhibition are distributed evenly across multiple TSSs. The TSS distributions for *isp5* (A), *per1* (B), *put4* (C), *spbpb2b2*.*01* (D) and *cat1* (E) are visualized by Integrative Genomics Viewer (IGV). The transcriptional start sites of *isp5* ^*+*^, *per1* ^*+*^, *put4* ^*+*^, *SPBPB2B2*.*01* and *cat1* ^*+*^ identified by CAGE are 25bp, 627bp, 140bp, 352bp and 359bp from their respective translational start sites according to PomBase. The upper half of each image shows the distribution of the TPM (transcripts per million) value along the chromosomal position at a single base resolution. Ticks appear at 10bp intervals along the horizontal axis. The TPM range is adjusted to visualize most peaks, so that some peaks above a TPM range may be truncated. Two images in (B) show the TSS distributions in two separate chromosomal regions upstream of the *per1* ^+^ gene with respective optimal TPM ranges. Two images in (C) show the TSS distribution in the same chromosomal region upstream of the *put4* ^+^ gene with different TPM range (0–150 for left; 0–10 for right). The lower half of each image shows the locations of top peaks (green) and bottom peaks (blue), which basically represent narrow and broad peaks, by horizontal bars. The fold change in logarithmic scale (base 2) relative to the value in wild-type cells is shown below respective bars. Positive and negative values indicate upregulation and downregulation, respectively, compared to wild-type cells.

**Fig 2:**
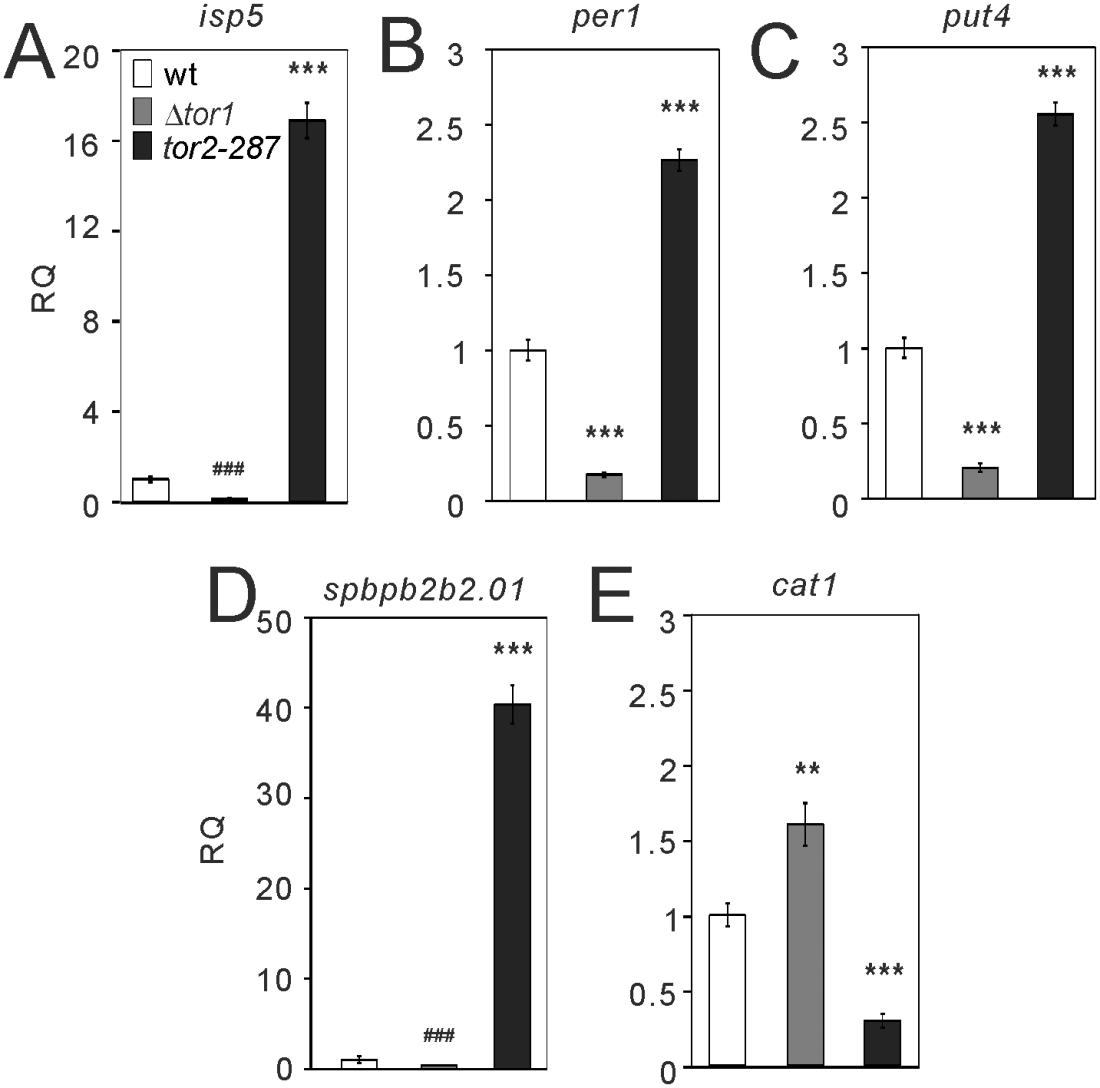
The loss of Tor1 and Tor2 inhibition oppositely affect mRNA expression of several amino acid permeases. Wild-type (wt) cells, Δ*tor1* cells and *tor2-287* cells were grown overnight in EMM medium to early log phase, and then the cells were harvested. Total RNA was extracted and subjected to semi-quantitative RT-PCR for the indicated amino acid permeases. The values were obtained by the comparative CT method in comparison to those of *act1*, and then were normalized to those in wild-type cells (RQ: relative quantity). The values were averaged from three independent experiments and are shown. ** *P*<0.01 and *** *P*<0.001 for Turkey’s test following one-way ANOVA for the comparisons with respective values of wild-type cells. ^###^ *P*<0.001 for unpaired *t*-test for the planned comparisons with respective values of wild-type cells.

Using CAGE technology, we obtained and compared the capped mRNA expression profiles of amino acid permeases in Δ*tor1* cells and *tor2-287* temperature-sensitive mutant cells with those in wild-type cells. In immunoblot analysis using Rps6 phosphorylation as a readout, *tor2-287* mutant cells showed reduced Tor2 activity at 27°C to avoid heat inducible gene expression, and this residual Tor2 activity was abolished at 34°C (S1 Fig). In this analysis, we used the mRNA samples from the cells cultured at 27°C, and identified up-regulated and down-regulated transcriptional start sites (TSSs) in *tor2-287* cells or Δ*tor1* cells, compared with wild-type cells (S1 and S2 Tables). Twenty-two amino acid permeases were significantly expressed in any one of the analyzed cells, and each amino acid permease showed distinct regulations by Tor2 and Tor1 (S3 Table). In *tor2-287* cells, 5 genes (*isp5* ^*+*^, *SPBPB2B2*.*01*, *put4* ^*+*^, *per1* ^*+*^, *SPCPB1C11*.*02*) were up-regulated, and 2 genes (*cat1* ^*+*^, *SPCC74*.*04*) were down-regulated, by at least 2 fold. In Δ*tor1* cells, 2 genes (*cat1* ^*+*^, *SPCPB1C11*.*02*) were up-regulated, and 7 genes (*isp5* ^*+*^, *SPBPB2B2*.*01*, *put4* ^*+*^, *per1* ^*+*^, *SPBPB10D8*.*01*, *SPCPB1C11*.*03*, *SPCC74*.*04*) were down-regulated, by at least 2 fold. Notably, Tor1 and Tor2 oppositely regulate the expression of several amino acid permeases: Tor1 increases, and Tor2 decreases, the expression levels of 4 amino acid permeases *(isp5* ^*+*^, *SPBPB2B2*.*01*, *put4* ^*+*^, and *per1* ^*+*^), whereas Tor1 decreases, and Tor2 increases, the expression level of *cat1* ^*+*^. Then we analyzed the distribution of TSSs differentially used in Δ*tor1* cells and *tor2-287* mutant cells, compared with wild-type cells (S4 Table). The opposite regulations by the loss of Tor1 and Tor2 inhibition appear to be evenly distributed across multiple TSSs in each of the genes we selected (*isp5*, *per1*, *put4*, *spbpb2b2*.*01* and *cat1*; Fig 1). Since the small sample size of our CAGE data precludes statistical analyses, all the above changes by the loss of Tor1 and Tor2 inhibition found by CAGE were confirmed to be statistically significant in semi-quantitative RT-PCR analysis with the larger sample size (Fig 2). These results indicate that Tor1 and Tor2 oppositely regulate transcription from the same TSSs in the gene of each amino acid permease.

#### Tor1 and Tor2 oppositely regulate transcriptional activity of *isp5* ^+^ promoter

**Fig 3:**
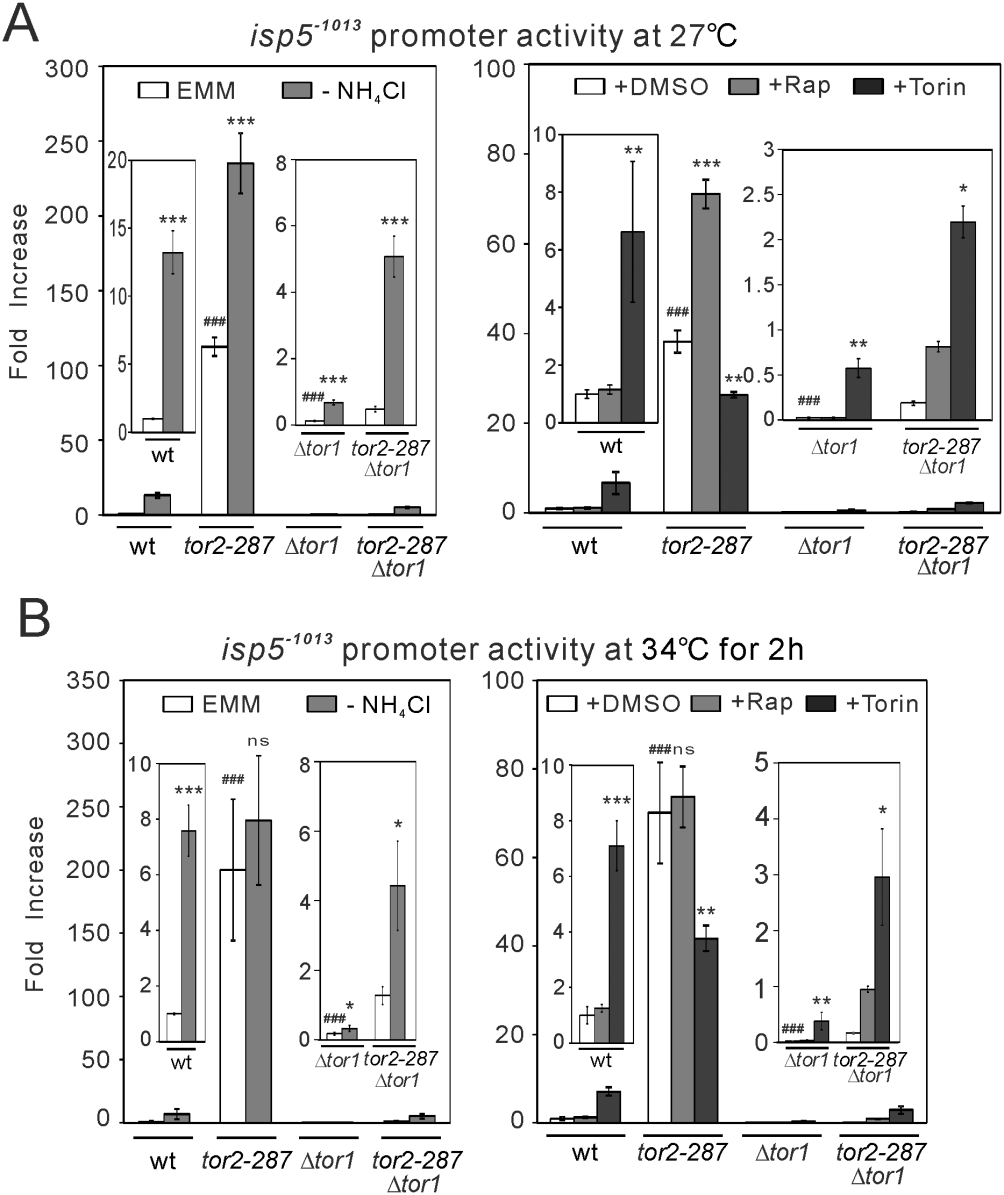
The loss of Tor1 decreases, and Tor2 inhibition and nitrogen depletion increases, *isp5* ^+^ promoter activity. Wild-type (wt), *tor2-287*, Δ*tor1* and *tor2-287*Δ*tor1* cells harboring the reporter plasmid for the 1013bp region of *isp5* ^+^ promoter (*isp5* ^-1013^; pKB8527) were grown to exponential phase at 27°C without (A) or with (B) shift to 34°C for 2 hours. The medium was then replaced by EMM or nitrogen-depleted EMM (-NH_4_Cl) (left graphs in A and B), or by EMM containing DMSO (+DMSO), 0.2 μg/ml rapamycin (+Rap) or 2 μM Torin-1 (+Torin) (right graphs in A and B), and the cells were subjected to Renilla luciferase reporter assay. The bioluminescence was measured real-time as relative light unit (RLU) for 2 hours, and the peak RLU value was normalized to that in wild-type cells in control conditions (EMM or +DMSO). The values were averaged from three independent experiments, and are shown. Magnified parts of the graphs are shown in insets. * *P*<0.05, ** *P*<0.01, *** *P*<0.001 for Tukey’s multiple comparison test following one-way ANOVA or unpaired *t*-test for the comparisons with vehicle conditions of respective genotypes. ^###^ *P*<0.001 for unpaired *t*-test for the comparison with vehicle conditions in wild-type cells. ns, not significant.

To confirm that Tor signaling regulates the promoter activity of amino acid permeases, we performed the Renilla luciferase reporter assay with the promoter of *isp5* ^+^ as a representative amino acid permease regulated by Tor signaling. The *isp5* ^+^ promoter used in this study covers the promoter region from the TSS to its 1013bp upstream (*isp5* ^-1013^). The basal activity of *isp5* ^+^ promoter was higher in *tor2-287* mutant cells, and was lower in Δ*tor1* cells, compared with wild-type cells at both 27°C and 34°C (Fig 3). Nitrogen depletion, which is known to inhibit Tor2 [6, 7], increased the activity of *isp5* ^+^ promoter in wild-type cells. Under nitrogen depletion, *tor2-287* mutant cells showed higher activity than wild-type cells at 27°C and 34°C. However, since the basal transcriptional activity was elevated in *tor2-287* mutant cells, nitrogen depletion-induced increase became smaller than that in wild-type cells at 27°C, and was abolished at 34°C, suggesting a critical role of Tor2 in nitrogen depletion-induced *isp5* ^*+*^ transcription. These results suggest that basal Tor2 activity inhibits the activity of *isp5* ^+^ promoter, and that nitrogen depletion disinhibits the *isp5* ^+^ promoter through inhibiting Tor2. By contrast, in Δ*tor1* cells, the basal activity and the nitrogen depletion-induced activation of the *isp5* ^+^ promoter were abolished (Fig 3), suggesting a critical role of Tor1 in the activity of the *isp5* ^+^ promoter. The activity of the *isp5* ^+^ promoter still requires Tor1 even when TORC1 activity is reduced, because the loss of Tor1 abolished both the basal transcriptional activity and nitrogen depletion-induced transcriptional activation in *tor2-287* cells (Fig 3).

We also examined the effects of representative Tor inhibitors on the *isp5* ^+^ promoter activity. Rapamycin, a Tor2-selective inhibitor [26, 27], did not affect the *isp5* ^+^ promoter activity in wild-type cells. However, in *tor2-287* mutant cells at 27°C with reduced Tor2 activity, rapamycin increased the *isp5* ^+^ promoter activity (Fig 3A). This rapamycin-induced transcriptional activation was abolished after shift to 34°C for 2 hours (Fig 3B). Therefore, rapamycin appears to induce *isp5* ^*+*^ transcription in a Tor2-dependent manner, although Tor2 activity in wild-type cells appears to be resistant to rapamycin treatment. Unlike rapamycin, Torin-1, an inhibitor for both Tor1 and Tor2 [28], increased the transcriptional activity of *isp5* ^+^ promoter in wild-type cells (Fig 3). By contrast, in *tor2-287* cells, Torin-1 treatment reduced the *isp5* ^+^ promoter activity from the level with vehicle treatment at both 27°C and 34°C, suggesting that this inhibitory effect of Torin-1 in *isp5* ^+^ promoter activity is independent from Tor2. Since Tor1 is critical for the *isp5* ^+^ promoter activity, as described above, the inhibitory effect of Torin-1 could be due to Tor1 inhibition.

#### The GATAAG motif is required for *isp5* ^+^ promoter activity

**Fig 4:**
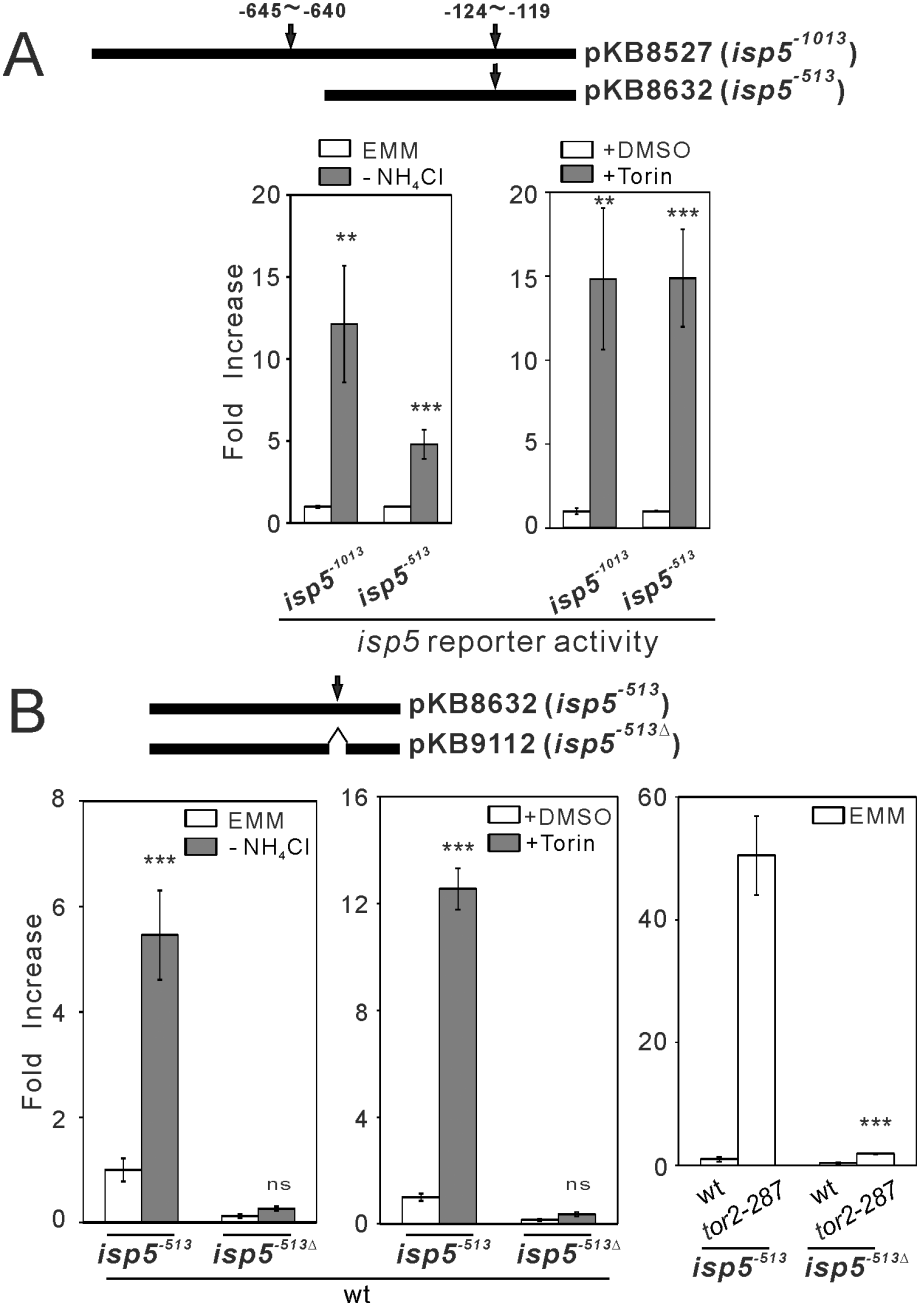
The GATAAG motif is necessary for *isp5* ^+^ promoter activity and its activation by Tor2 inhibition and nitrogen depletion. (A) The 1013-bp region (*isp5* ^-1013^) and the 513bp region (*isp5* ^-513^) of *isp5* ^+^ promoter from the TSS were used to generate Renilla luciferase reporters (pKB8527 and pKB8632, respectively). The *isp5* ^-1013^ promoter region contains two GATAAG motifs at designated locations (-645~-640bp and -124~-119bp), and the *isp5* ^-513^ promoter region contains only one of these motifs proximal to the TSS. Both of these two GATAAG motifs (arrows) are on the complement strand of the *isp5* ^+^ promoter. Wild-type cells harboring the respective reporter plasmids were grown to exponential phase at 27°C, and were assayed without or with nitrogen depletion (EMM and -NH_4_Cl, respectively) or without or with Torin-1 treatment (+DMSO and +Torin, respectively). The bioluminescence was measured and analyzed as described in Fig 3. The data were obtained from three independent experiments. ** *P*<0.01, *** *P*<0.001 for unpaired *t*-test for the comparisons with respective vehicle conditions. (B) The 513bp region of *isp5* ^+^ promoter (*isp5* ^-513^) and the same promoter region with the GATAAG motif deleted (*isp5* ^-513Δ^) were used to generate Renilla luciferase reporters (pKB8632 and pKB9112, respectively). For the left two graphs, wild-type (wt) cells harboring the respective reporter plasmids were grown to exponential phase, and were assayed without or with nitrogen depletion (EMM and -NH_4_Cl, respectively) or without or with Torin-1 treatment (+DMSO and +Torin, respectively). For the rightmost graph, wild-type (wt) cells and *tor2-287* cells harboring the respective reporter plasmids were analyzed without stimulation (EMM). The bioluminescence was measured and analyzed as described in Fig 3. The data were obtained from three independent experiments. *** *P*<0.001 for unpaired *t*-test for respective vehicle conditions. ns, not significant.

To identify potential transcription factors involved in Tor-mediated regulation of gene expression, we performed the motif discovery analysis [14] using our CAGE results. The TSSs with at least 2-fold changes in *tor2-287* or Δ*tor1* cells were classified into 4 groups; up-regulated at top peaks, down-regulated at top peaks, up-regulated at bottom peaks, and down-regulated at bottom peaks. The top 100 TSSs with the highest concentration in each group were separately analyzed. We identified that GATA motifs are overrepresented in the promoter regions associated with the TSSs up-regulated at top and bottom peaks as well as those down-regulated at top peaks in *tor2-287* cells. GATA transcription factors are a family of transcription factors characterized by their ability to bind to the DNA sequence “GATA” [29]. The GATAAG motif is one of the sequence motifs to which GATA transcription factors are known to bind [30, 31]. In the *isp5* ^+^ promoter, there are two GATAAG motifs located at the -645~-640bp region (distal) and the -124~-119bp region (proximal) upstream of the TSS (Fig 4A). The distal GATAAG motif was not critical for *isp5* ^+^ promoter activity, since the shorter *isp5* ^+^ promoter (*isp5* ^-513^) lacking this motif showed the normal basal activity of *isp5* ^+^ promoter and its activation induced by nitrogen depletion and Torin-1 treatment (Fig 4A). To determine the importance of the proximal GATAAG motif, we selectively removed this motif from the *isp5* ^-513^ promoter. The resulting promoter lacking the GATAAG motif (*isp5* ^-513Δ^) showed negligible basal activity and the lack of activation induced by nitrogen depletion, Torin-1 treatment or Tor2 inhibition (Fig 4B). These results suggest that the proximal GATAAG motif at the -124~-119bp region from the TSS is critical for the basal activity of *isp5* ^+^ promoter and its activation induced by Tor2 inhibition and nitrogen depletion.

#### Tor1 and Tor2 oppositely regulate GATAAG-mediated transcription

**Fig 5:**
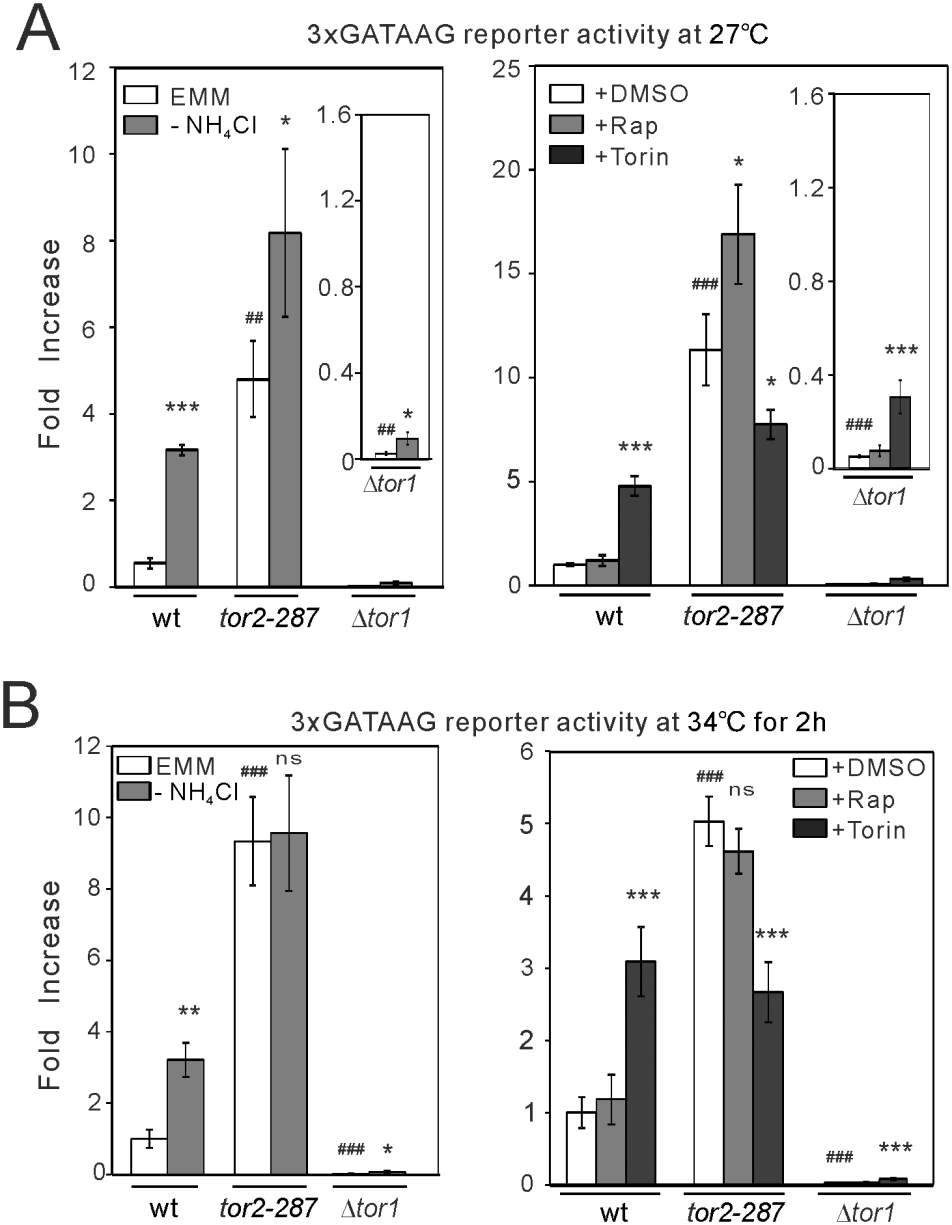
The loss of Tor1 decreases, and Tor2 inhibition and nitrogen depletion increases, the activity of the GATAAG reporter. Wild-type (wt) cells, *tor2-287* cells and Δ*tor1* cells harboring the luciferase reporter for the GATAAG motif (3xGATAAG reporter; pKB8742) were grown to exponential phase at 27°C without (A) or with (B) shift to 34°C for 2 hours. The cells were assayed without or with nitrogen depletion (EMM and -NH_4_Cl, respectively) or without or with rapamycin or Torin-1 treatment (+DMSO, +Rap, +Torin, respectively). The bioluminescence was measured and analyzed as described in Fig 3. The data were obtained from three independent experiments. Magnified parts of the graphs are shown in insets. * *P*<0.05, ** *P*<0.01, *** *P*<0.001 for Tukey’s multiple comparison test following one-way ANOVA or unpaired *t*-test for the comparisons with vehicle conditions of respective genotypes. ^##^ *P*<0.01, ^###^ *P*<0.001 for unpaired *t*-test for the comparisons with vehicle conditions in wild-type cells. ns, not significant.

We then examined whether Tor1 and Tor2 regulate transcription mediated by the GATAAG motif, using its Renilla luciferase reporter with the promoter containing three tandem copies of this motif (3xGATAAG). This GATAAG reporter recapitulated the patterns of Tor-mediated regulations of the *isp5* ^+^ promoter, as follows. In wild-type cells, nitrogen depletion increased the activity of GATAAG reporter (Fig 5). Under nitrogen depletion, *tor2-287* mutant cells showed higher activity of GATAAG reporter than wild-type cells at 27°C and 34°C. However, since the basal transcriptional activity was elevated in *tor2-287* mutant cells, a relative increase induced by nitrogen depletion became smaller than in wild-type cells at 27°C, and was abolished at 34°C, in which Tor2 activity was fully suppressed. These results suggest that basal Tor2 activity suppresses GATAAG-mediated transcription, and that nitrogen depletion disinhibits this transcription through inhibiting Tor2. By contrast, in Δ*tor1* cells, the basal activity of the GATAAG reporter and its activation induced by nitrogen depletion were abolished (Fig 5). It is noted that the loss of Tor1 did not affect the activity of a reporter for the CDRE motif (S2 Fig). Therefore, Tor1 is selectively involved in GATAAG-mediated transcription.

We also examined the effects of rapamycin and Torin-1 on GATAAG reporter activity. The GATAAG reporter also recapitulated the effects of these drugs observed with *isp5* ^+^ promoter. Thus, rapamycin did not increase GATAAG reporter activity in wild-type cells. However, rapamycin significantly increased this reporter activity in *tor2-287* cells at 27°C (Fig 5A). This rapamycin-induced activation was abolished at 34°C (Fig 5B), in which Tor2 activity is fully suppressed. Therefore, rapamycin appears to induce GATAAG-mediated transcription in a manner dependent on Tor2, although rapamycin alone may not suppress Tor2 activity in wild-type cells. By contrast, Torin-1 treatment increased GATAAG reporter activity in wild-type cells, whereas it decreased the reporter activity in *tor2-287* mutant cells at both 27°C and 34°C (Fig 5), indicating that the inhibitory effect of Torin1 is independent from Tor2. Since Tor1 is critical for GATAAG-mediated transcription, Torin-1-induced suppression could be due to Tor1 inhibition.

#### Gaf1 is critical for transcription driven by *isp5* ^+^ promoter and the GATAAG motif

**Fig 6:**
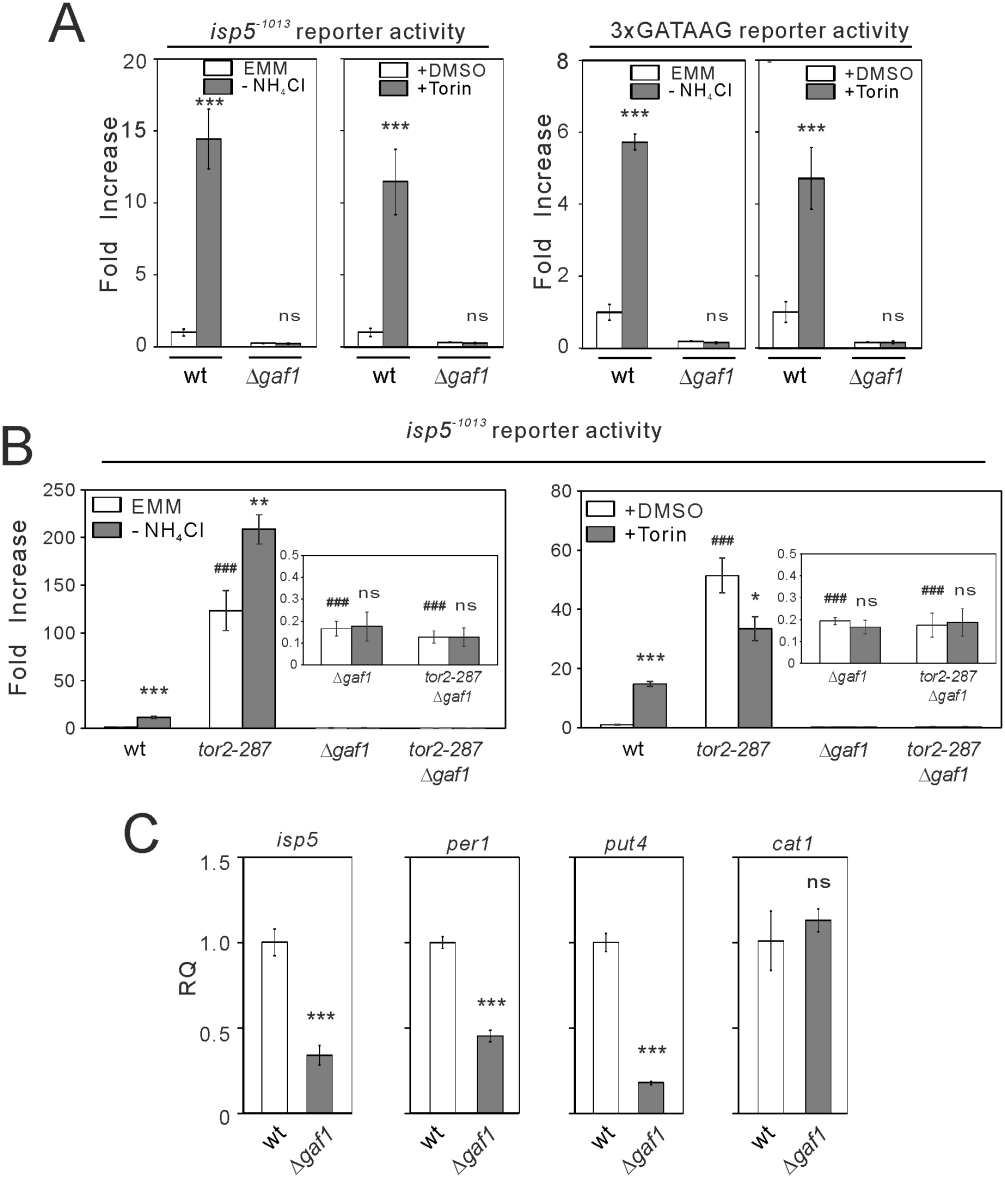
Gaf1 is critical for the transcription driven by *isp5* ^+^ promoter and the GATAAG motif. (A) The loss of the basal activities of *isp5* ^+^ promoter and the GATAAG reporter and their activation by nitrogen depletion and Torin-1 treatment in Δ*gaf1* cells. Wild-type (wt) cells and Δ*gaf1* cells harboring the reporter plasmids for *isp5* ^+^ promoter (*isp5* ^-1013^; pKB8527) or the GATAAG motif (3xGATAAG; pKB8742) were grown to exponential phase and assayed without or with nitrogen depletion (EMM or -NH_4_Cl, respectively) or without or with Torin-1 treatment (+DMSO or +Torin, respectively). The bioluminescence was measured and analyzed as described in Fig 3. The data were obtained from three independent experiments. *** *P*<0.001 for Tukey’s multiple comparison test following one-way ANOVA or unpaired *t*-test for the comparisons with vehicle conditions of respective genotypes. ns, not significant. (B) The loss of Gaf1 abolished the basal activity of *isp5* ^+^ promoter and its activation by Tor2 inhibition. Wild-type (wt) cells, *tor2-287* cells, Δ*gaf1* cells and *tor2-287*Δ*gaf1* cells harboring the reporter plasmid for *isp5* ^+^ promoter (*isp5* ^-1013^) were grown to exponential phase, and were assayed without or with nitrogen depletion (EMM or -NH_4_Cl, respectively) or without or with Torin-1 treatment (+DMSO or +Torin, respectively). The bioluminescence was measured and analyzed as described in Fig 3. The data were obtained from three independent experiments. Magnified parts of the graphs are shown in insets. * *P*<0.05, ** *P*<0.01, *** *P*<0.001 for Tukey’s multiple comparison test following one-way ANOVA or unpaired *t*-test for the comparisons with vehicle conditions of respective genotypes. ^###^ *P*<0.001 for unpaired *t*-test for the comparisons with vehicle values in wild-type cells. ns, not significant. (C) Reduced mRNA levels of *isp5*, *per1*, *put4*, but not *cat1*, in Δ*gaf1* cells. Wild-type (wt) cells and Δ*gaf1* cells were grown overnight in EMM media to early log phase. Total RNA was extracted and subjected to semi-quantitative RT-PCR analysis. The data were obtained from three independent experiments. *** *P*<0.001 for Tukey’s multiple comparison test following one-way ANOVA or unpaired *t*-test for the comparisons with vehicle conditions of respective genotypes. ns, not significant.

In fission yeast, there are five GATA transcription factors named Sfh1/SPCC16A11.14, Fep1/SPAC23E2.01, Ams2/SPCC290.04, Gaf1/SPCC1902.01, and SPCC1393.08 (http://www.pombase.org/spombe/related/PBO:0000371). We performed transcriptional activity of the *isp5* ^+^ promoter and the GATAAG reporter in Δ*gaf1*, Δ*fep1* and Δ*SPCC1393*.*08* cells, three strains available from the whole-genome knockout library [17]. In Δ*gaf1* cells, the basal activities of these reporters and their activation induced by nitrogen depletion and Torin-1 treatment were abolished (Fig 6A). The other two strains appeared to show normal activities of these reporters (data not shown). The loss of Gaf1 also abolished the elevated basal activity of *isp5* ^+^ promoter in *tor2-287* mutant cells (Fig 6B). These findings show that Gaf1 is critical for the basal activity of *isp5* ^+^ promoter as well as its activation induced by nitrogen depletion and Tor2 inhibition. Furthermore, the mRNA levels of *isp5*, *per1* and *put4*, but not *cat1*, were significantly decreased in Δ*gaf1* cells (Fig 6C), suggesting a universal role of Gaf1 in transcription of multiple amino acid permeases.

#### Tor2 inhibition induces nuclear localization of Gaf1 and its dephosphorylation

**Fig 7:**
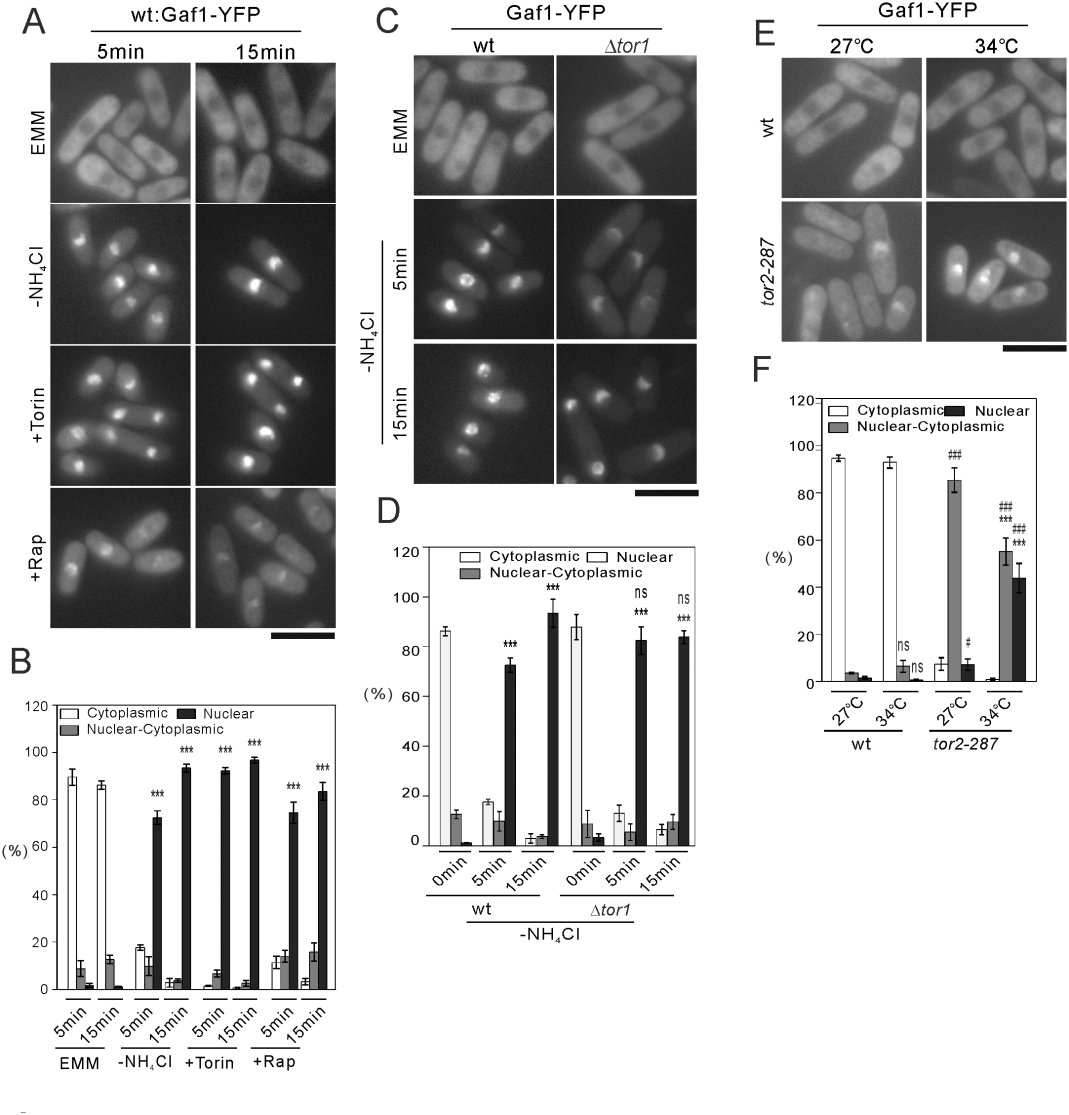
Tor2 inhibition and nitrogen depletion induce nuclear localization of Gaf1. (A) Nuclear localization of Gaf1 induced by nitrogen depletion and Tor inhibitors. Wild-type (wt) cells expressing Gaf1-YFP under its native promoter were grown to early log phase in EMM medium at 27°C. The cells were incubated without (EMM) or with nitrogen depletion (-NH_4_Cl), Torin-1 treatment (+Torin) or rapamycin treatment (+Rapamycin) for 5 or 15 min, and the fluorescent images of Gaf1-YFP were then acquired. Scale bar, 10 μm. (B) Quantification of nuclear localization of Gaf1 in the experiments shown in (A). The proportions of the cells which showed Gaf1 signals in the nucleus alone (Nuclear), in the cytosol alone (Cytoplasmic), or in both (Nuclear-Cytoplasmic) were determined. The averaged values from 3 independent experiments are shown. *** *P*<0.001 for Tukey’s multiple comparison test following one-way ANOVA for the comparisons with EMM condition at respective time points. (C) Intact nuclear localization of Gaf1 induced by nitrogen depletion in Δ*tor1* cells. Δ*tor1* cells expressing Gaf1-YFP under its native promoter were grown to early log phase in EMM media at 27°C. The cells were incubated before (EMM) or after nitrogen depletion (-NH_4_Cl) for 5 or 15 min, and the fluorescent images of Gaf1-YFP were then acquired. Scale bar, 10 μm. (D) Quantification of nuclear localization of Gaf1 in the experiments shown in (C). The proportions of the cells which showed Gaf1 signals in the nucleus alone, in the cytosol alone, or in both were determined. The averaged values from 3 independent experiments are shown. ***P<0.001 for Tukey’s multiple comparison test following one-way ANOVA for the comparisons with control conditions of respective genotypes. ns, not significant for unpaired *t*-test for the comparisons with corresponding values in wild-type cells. (E) Nuclear localization of Gaf1 induced by Tor2 inhibition. Wild-type (wt) cells and *tor2-287* cells expressing Gaf1-YFP under its native promoter were grown to early log phase in EMM media at 27°C. A half of the cells were maintained at 27°C, and the other half were cultured at 34°C for 2 hours. The fluorescent images of Gaf1-YFP were then acquired. Scale bar, 10 μm. (F) Quantification of nuclear localization of Gaf1 in the experiments shown in (E). The proportions of the cells which showed Gaf1 signals in the nucleus alone, in the cytosol alone, or in both were determined. The averaged values from 3 independent experiments are shown. ***P<0.001 and ns, not significant for Tukey’s multiple comparison test following one-way ANOVA for the comparisons with the values at 27°C. ^#^ *P*<0.05, ^###^ *P*<0.001 for Tukey’s multiple comparison test following one-way ANOVA for the comparison with corresponding values in wild-type cells.

To investigate how Gaf1 is regulated for transcription of amino acid permeases, we observed the localization of Gaf1-YFP expressed under its native promoter. Since expression of Gaf1-YFP rescued defective *isp5* ^*+*^ expression in Δ*gaf1* cells (S3 Fig), Gaf1-YFP is functional. In wild-type cells cultured in the EMM medium, Gaf1-YFP was mainly localized to the cytosol (Fig 7A and 7B and S4 Fig). Nitrogen depletion and Torin-1 treatment induced robust nuclear localization of Gaf1-YFP from the cytosol as early as 5 min (Fig 7A and 7B). Although rapamycin treatment alone did not induce transcription of amino acid permeases, as previously reported [9, 27] and shown in this study, it also induced nuclear localization of Gaf1-YFP, albeit to the much lesser extent. We then investigated the involvement of Tor1 and Tor2 in nuclear localization of Gaf1. In Δ*tor1* cells, Gaf1-YFP was localized to the cytosol in the EMM medium, and was localized to the nucleus upon nitrogen depletion (Fig 7C and 7D). However, nuclear signals of Gaf1-YFP appear to be weaker in Δ*tor1* cells, compared with wild-type cells (Fig 7C). The *tor2-287* cells showed abnormal localization of Gaf1. Gaf1 was slightly enriched in the nucleus at 27°C, and was completely localized to the nucleus at 34°C (Fig 7E and 7F). Therefore, basal Tor2 activity suppresses, and Tor2 inhibition and nitrogen depletion induces, nuclear localization of Gaf1.

**Fig 8:**
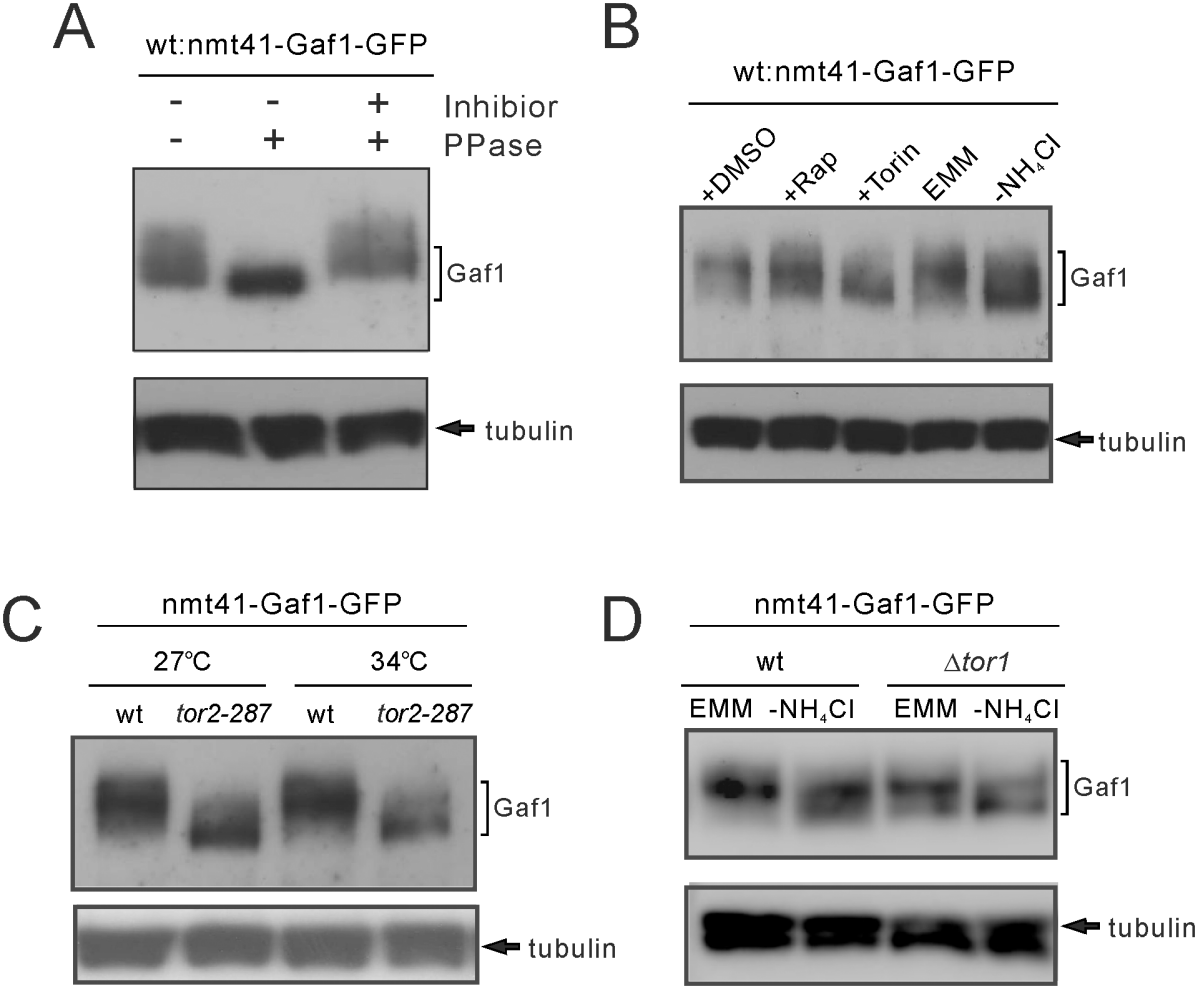
Tor2 inhibition and nitrogen depletion induce Gaf1 dephosphorylation. (A) Immunoblot analysis of Gaf1 phosphorylation. To induce Gaf1-GFP expression, wild-type (wt) cells expressing Gaf1-GFP under the *nmt41* promoter were grown to early log phase in EMM medium without thiamine at 27°C for 20 hours. Proteins were extracted and treated without or with λ-phosphatase (PPase) in the presence or absence of its inhibitors, as indicated in the graph. The resultant proteins were subjected to SDS-PAGE and immunoblot analyses with anti-GFP antibodies. Endogenous α-tubulin was detected as a loading control. (B) Gaf1 dephosphorylation induced by Torin1 treatment and nitrogen depletion. To induce Gaf1-GFP expression, wild-type (wt) cells expressing Gaf1-GFP under the control of the *nmt41* promoter were grown to early log phase in EMM media without thiamine at 27°C for 20 hours. The cells were treated without or with rapamycin or Torin-1 (+DMSO, +Rap or +Torin, respectively) or without or with nitrogen depletion (EMM or -NH_4_Cl, respectively) for 15 min. The lysates of these cells were analyzed similarly to (A). (C) Gaf1 dephosphorylation induced by Tor2 inhibition. To induce Gaf1-GFP expression, wild-type (wt) cells and *tor2-287* cells expressing Gaf1-GFP under the *nmt41* promoter were grown to early log phase in EMM medium without thiamine at 27°C for 20 hours. The lysates of these cells were analyzed similarly to (A). (D) Intact Gaf1 dephosphorylation induced upon nitrogen depletion in Δ*tor1* cells. To induce Gaf1-GFP expression, wild-type (wt) cells and Δ*tor1* cells expressing Gaf1-GFP under the *nmt41* promoter were grown to early log phase in EMM medium without thiamine at 27°C for 20 hours. The lysates of these cells were analyzed similarly to (A).

It has been reported that dephosphorylation of Gln3, a GATA transcription factor in budding yeast, occurs simultaneously with its nuclear localization [32, 33]. We examined whether Gaf1 phosphorylation is also regulated by nitrogen depletion and Tor2 inhibition in fission yeast. We performed immunoblot analysis using Gaf1-GFP expressed under the *nmt41* promoter to examine Gaf1 phosphorylation (Fig 8A). Since expression of Gaf1-GFP rescued defective *isp5* ^*+*^ expression in Δ*gaf1* cells (S3 Fig), Gaf1-GFP is functional. In this analysis, Gaf1 signals were broadly distributed around the expected molecular weight. λ-phosphatase treatment increased the mobility of Gaf1 signals, so that the signals formed a sharper band. This effect of λ-phosphatase was not observed in the presence of its inhibitors. These results suggest that the mobility shift of Gaf1 signals in immunoblot analysis is due to Gaf1 phosphorylation. Torin-1 treatment and nitrogen depletion increased the mobility of Gaf1, whereas rapamycin treatment appeared to have no effect on Gaf1 mobility (Fig 8B). In *tor2-287* mutant cells, Gaf1 mobility was also faster than in wild-type cells at both 27°C and 34°C (Fig 8C). In Δ*tor1* cells, nitrogen depletion induced faster Gaf1 mobility similarly to wild-type cell (Fig 8D). These findings suggest that nitrogen depletion and Tor2 inhibition, but not Tor1, induces Gaf1 dephosphorylation along with its nuclear localization.

## Discussion

Tor signaling is critical for transcriptional regulation of amino acid permeases according to nitrogen availability. However, the underlying mechanism remains largely unknown. Here we have found that the loss of Tor1 decreases, and Tor2 inhibition increases, transcription of *isp5* ^*+*^, *per1* ^*+*^, *put4* ^*+*^ and *SPBPB2B2*.*01*, whereas the loss of Tor1 increases, and Tor2 inhibition decreases, transcription of *cat1* ^*+*^. These opposite regulations by Tor1 and Tor2 occur evenly across multiple TSSs identified by CAGE for respective genes of amino acid permeases. In luciferase reporter assay, nitrogen depletion and Tor2 inhibition induce the activation of *isp5* ^+^ promoter, and Tor1 is critical for the basal activity of *isp5* ^+^ promoter. The basal activity and Tor-mediated regulation of *isp5* ^+^ promoter depend on one of its GATAAG motifs, and the GATAAG reporter recapitulates Tor-mediated regulations of *isp5* ^+^ promoter. Furthermore, a GATA transcription factor Gaf1 is critical for the basal transcription of *isp5* ^*+*^ and its activation upon nitrogen depletion and Tor2 inhibition. Therefore, this study substantiates a critical role of Tor-Gaf1 signaling in transcriptional regulation of several amino acid permeases.

Since the GATAAG motif in *isp5* ^+^ promoter is critical for its transcriptional activity, it is plausible that Gaf1 binds to this motif for *isp5* ^*+*^ transcription, although the possibility that Gaf1 might indirectly regulate *isp5* ^*+*^ transcription cannot be excluded. Consistent with the role of Gaf1, nitrogen depletion and Tor2 inhibition induce nuclear localization of Gaf1 and its dephosphorylation. These regulations of Gaf1 have also been reported by another group recently [10]. Similar to these findings, in budding yeast, inhibition of TOR1, a core kinase of TORC1 in this organism, also leads to nuclear localization and dephosphorylation of Gln3, a GATA transcription factor [32, 33]. Nuclear localization of Gaf1 induced by nitrogen depletion and Tor2 inhibition could increase the concentration of Gaf1 in the nucleus, thereby promoting its binding to the GATAAG motif in *isp5* ^+^ promoter. Indeed, nitrogen starvation with proline as a poor quality nitrogen source also induces nuclear localization of Gaf1, and increases Gaf1 binding to the *isp7* ^+^ promoter containing three potential GATA motifs [34]. However, whether and how Gaf1 dephosphorylation contributes to its nuclear localization and Gaf1-mediated regulation of *isp5* ^*+*^ transcription remains to be elucidated. By contrast, since the loss of Tor1 abolishes *isp5* ^*+*^ transcription as well as GATAAG-mediated transcription, it is plausible that Tor1 promotes *isp5* ^*+*^ transcription through Gaf1 binding to the GATAAG motif in *isp5* ^+^ promoter in a manner opposite to Tor2. If that is the case, Tor1 regulates Gaf1 in a manner different from Tor2, since the loss of Tor1 affected neither nuclear localization nor phosphorylation of Gaf1. The mechanism about how Tor1 regulates Gaf1-mediated transcription remains to be investigated.

As discussed above, our findings suggest that nitrogen depletion suppresses Tor2 activity, thereby increasing *isp5* ^+^ transcriptional activity through Gaf1. However, *tor2-287* cells showed much higher activity of *isp5* ^+^ promoter than that in wild-type cells under nitrogen depletion. This difference cannot be explained by the magnitude of inhibition of Tor2 activity, because nitrogen depletion for 15 min abolishes Tor2 activity [24], whereas *tor2-287* cells showed reduced, but significantly Tor2 activity at the permissive temperature, as measured by Rps6 phosphorylation (S1 Fig). Since long-term partial suppression in Tor2 activity in *tor2-287* cells altered expression profiles of various genes (S1 Table), Tor2 may indirectly regulate *isp5* ^+^ transcriptional activity through some of these genes.

In addition to *isp5* ^*+*^ transcription, Tor1 and Tor2 also oppositely regulate mRNA expression of other amino acid permease genes, namely *per1* ^*+*^, *put4* ^*+*^ and *SPBPB2B2*.*01*. Gaf1 is also critical for mRNA expression of *isp5* ^*+*^, *per1* ^*+*^ and *put4* ^*+*^. These results suggest that Gaf1 coordinates transcription of multiple amino acid permeases upon Tor2 inhibition and nitrogen depletion. Although the GATAAG motif was not found in the promoters of *per1* ^+^ and *put4* ^+^, GATA-containing sequences similar to this motif exist. Whether these sequences mediate Gaf1-mediated transcription remains to be investigated. In contrast, Tor1 suppresses, and Tor2 promotes, the transcription of *cat1* ^*+*^ in a direction opposite to *isp5* ^*+*^, *per1* ^*+*^ and *put4* ^*+*^. Since the loss of Gaf1 did not affect the mRNA expression of *cat1*, another unidentified transcription factor must be involved. Therefore, Tor1 and Tor2 may regulate transcription of amino acid permeases in multiple ways through distinct transcription factors. Since each amino acid permease has its own substrate specificity and subcellular localization, this study also paves the way for elucidating the pattern of amino acid flux to promote cell proliferation and growth upon nutrient challenge.

## Supporting Information

S1 Fig Altered Tor2 activity in *tor2-287* cells. The wild-type (KP5080) and *tor2-287* (KP5734) cells were grown to exponential phase at 27°C without or with shift to 34°C for 2 hours. Proteins were extracted and subjected to SDS-PAGE and immunoblot analyses of Rps6 phosphorylation (P-Rps6) as a readout for Tor2 activity. Endogenous α-tubulin was detected as a loading control.
(TIF)

S2 Fig Transcriptional activity of the CDRE reporter is intact in Δ*tor1* cells. Wild-type (wt) cells and Δ*tor1* cells harboring CDRE Renilla reporter (pKB9132) were grown to exponential phase and assayed without or with extracellular Ca^2+^ stimulation (H_2_O or +100mMCaCl_2_, respectively). The bioluminescence was measured and analyzed as described in Fig 3. The data were obtained from three independent experiments. Note that the loss of Tor1 did not abolish the activity of the CDRE reporter and its Ca^2+^-induced activation, though it increased the CDRE reporter activity without stimulation.
(TIF)

S3 Fig The expression of either Gaf1-YFP or Gaf1-GFP proteins rescues defective *isp5* ^+^ expression in Δ*gaf1* cells. Wild-type (wt) cells harboring control vector, Δ*gaf1* cells harboring the control vector or the plasmids expressing Gaf1-GFP or Gaf1-YFP under the *nmt* promoter were grown overnight in EMM medium in the presence of 50 μM thiamine to early log phase, and then the cells were harvested. Total RNA was extracted and subjected to semi-quantitative RT-PCR for the indicated amino acid permeases. The values were obtained by the comparative CT method in comparison to those of *act1*, and then were normalized to those in wild-type cells (RQ: relative quantity). The values were averaged from three independent experiments and were shown. Note that weak, leaky expression of Gaf1-YFP or Gaf1-GFP in the presence of thiamine is sufficient to rescue defective *isp5* ^*+*^ expression in Δ*gaf1* cells.
(TIF)

S4 Fig Nitrogen depletion and Torin-1 treatment induce nuclear localization of Gaf1. Wild-type (wt) cells expressing Gaf1-YFP under its native promoter were grown to early log phase in EMM medium at 27°C. The cells were treated without or with nitrogen depletion or Torin1 treatment (EMM, -NH_4_Cl and +Torin, respectively) for 15 min. Nuclear staining with Hoechst demonstrates nuclear localization of Gaf1-YFP induced by nitrogen depletion and Torin-1 treatment. Scale bar, 10 μm.
(TIF)

S1 Table The list of TSSs that were up-regulated or down-regulated in *tor2-287* cells with log(2)FC≥1 or log(2)FC≤-1. (XLS)

S2 Table The list of TSSs that were up-regulated or down-regulated in Δ*tor1* cells with log(2)FC≥1 or log(2)FC≤-1. (XLS)

S3 Table The expression profiling of amino acid permeases with TPM (transcripts per million) ≥1 based on CAGE TSS clustering. (XLSX)

S4 Table The TSSs of amino acid permeases that were up-regulated or down-regulated in *tor2-287* or Δ*tor1* cells with log(2)FC≥1 or log(2)FC≤-1. (XLSX)
