# Supplementary figures and images for: Tor Signaling Regulates Transcription of Amino Acid Permeases through a GATA Transcription Factor Gaf1 in Fission Yeast
Source: PLoS One. 2015 Dec 21;10(12):e0144677. doi: 10.1371/journal.pone.0144677 (PMC4686964; doi:10.1371/journal.pone.0144677)

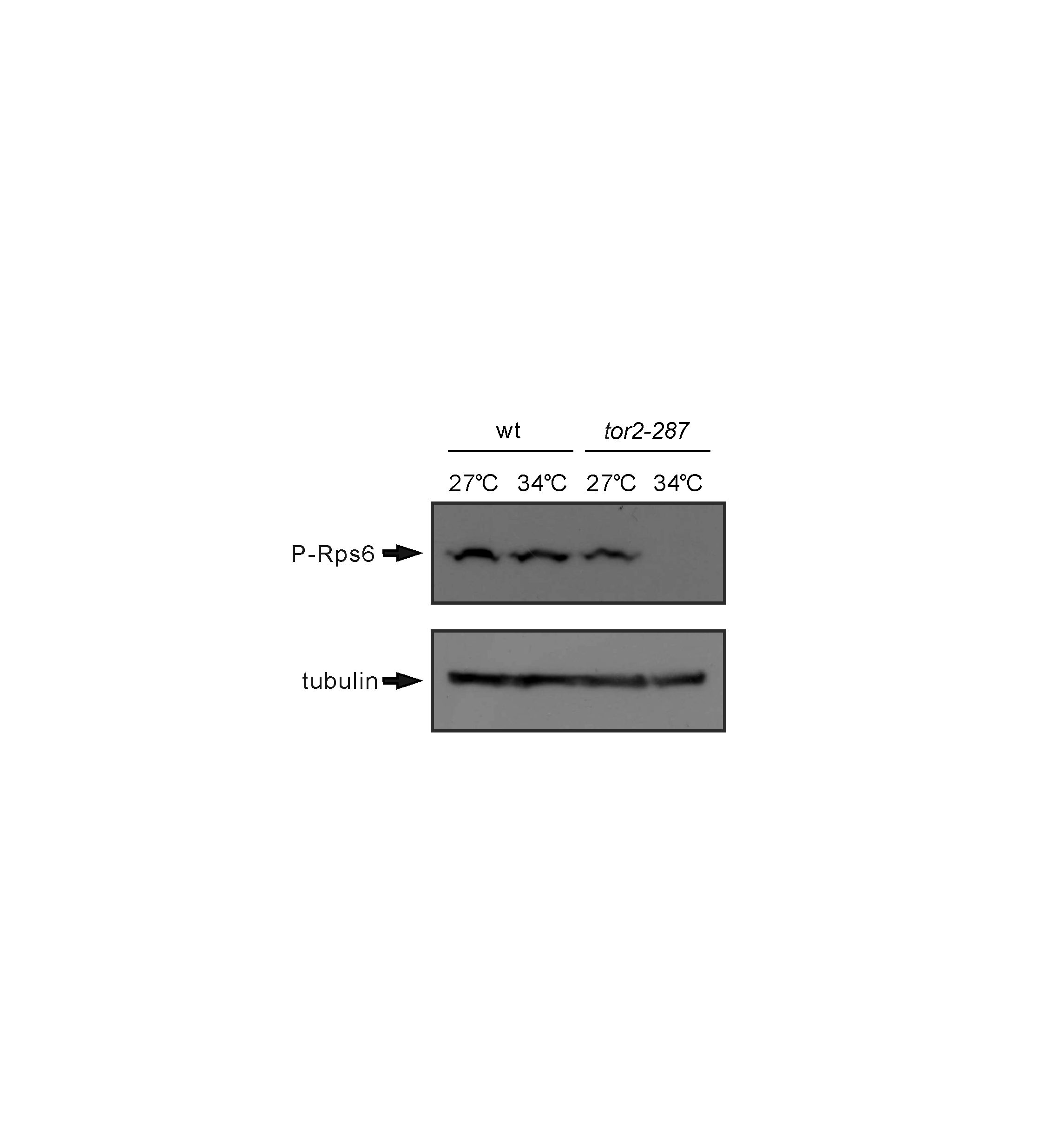

Supplement: S1 Fig — The wild-type (KP5080) and tor2-287 (KP5734) cells were grown to exponential phase at 27°C without or with shift to 34°C for 2 hours. Proteins were extracted and subjected to SDS-PAGE and immunoblot analyses of Rps6 phosphorylation (P-Rps6) as a readout for Tor2 activity. Endogenous α-tubulin was detected as a loading control. (TIF) [file pone.0144677.s001.tif]

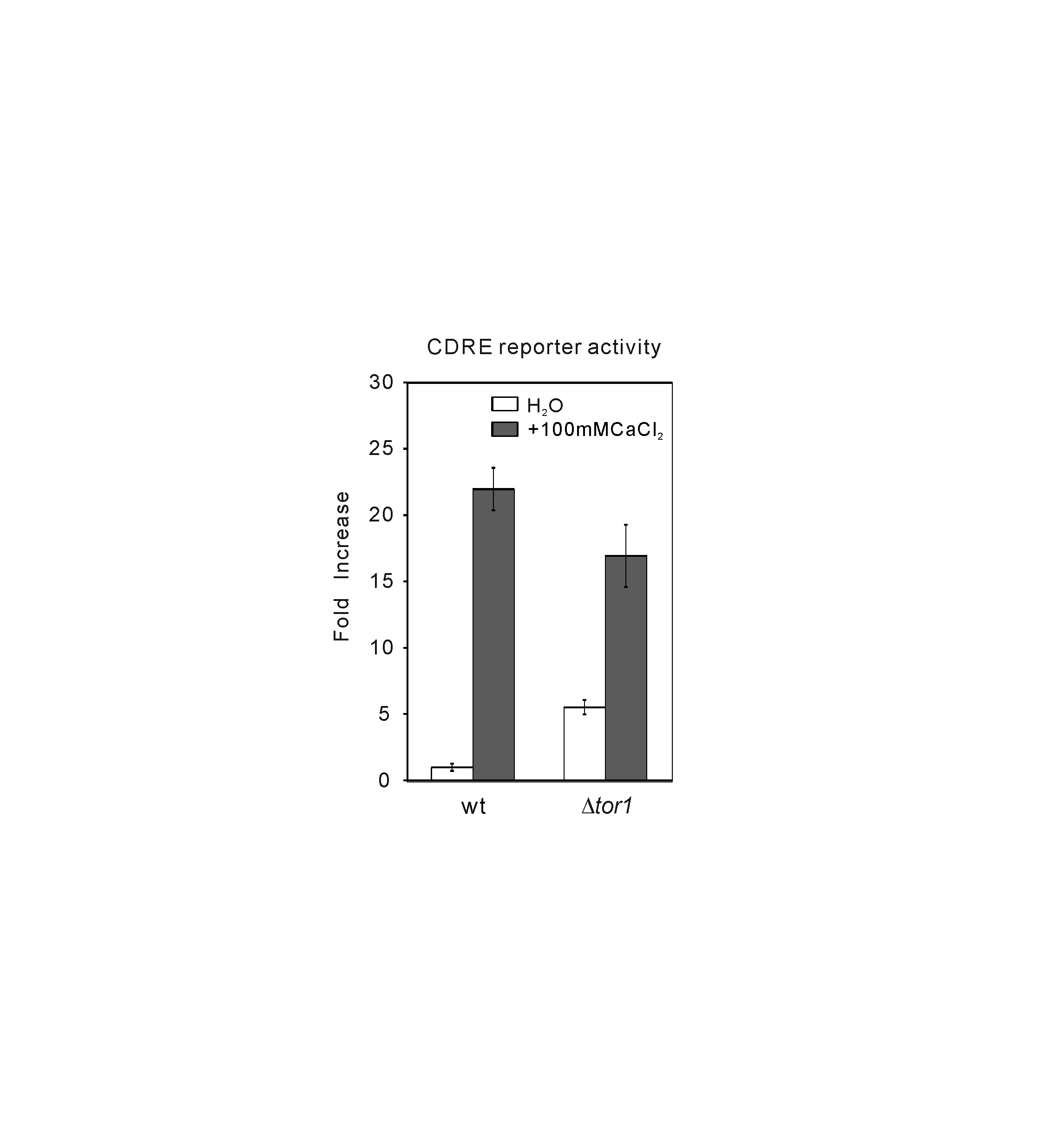

Supplement: S2 Fig — Wild-type (wt) cells and Δtor1 cells harboring CDRE Renilla reporter (pKB9132) were grown to exponential phase and assayed without or with extracellular Ca2+ stimulation (H2O or +100mMCaCl2, respectively). The bioluminescence was measured and analyzed as described in Fig 3. The data were obtained from three independent experiments. Note that the loss of Tor1 did not abolish the activity of the CDRE reporter and its Ca2+-induced activation, though it increased the CDRE reporter activity without stimulation. (TIF) [file pone.0144677.s002.tif]

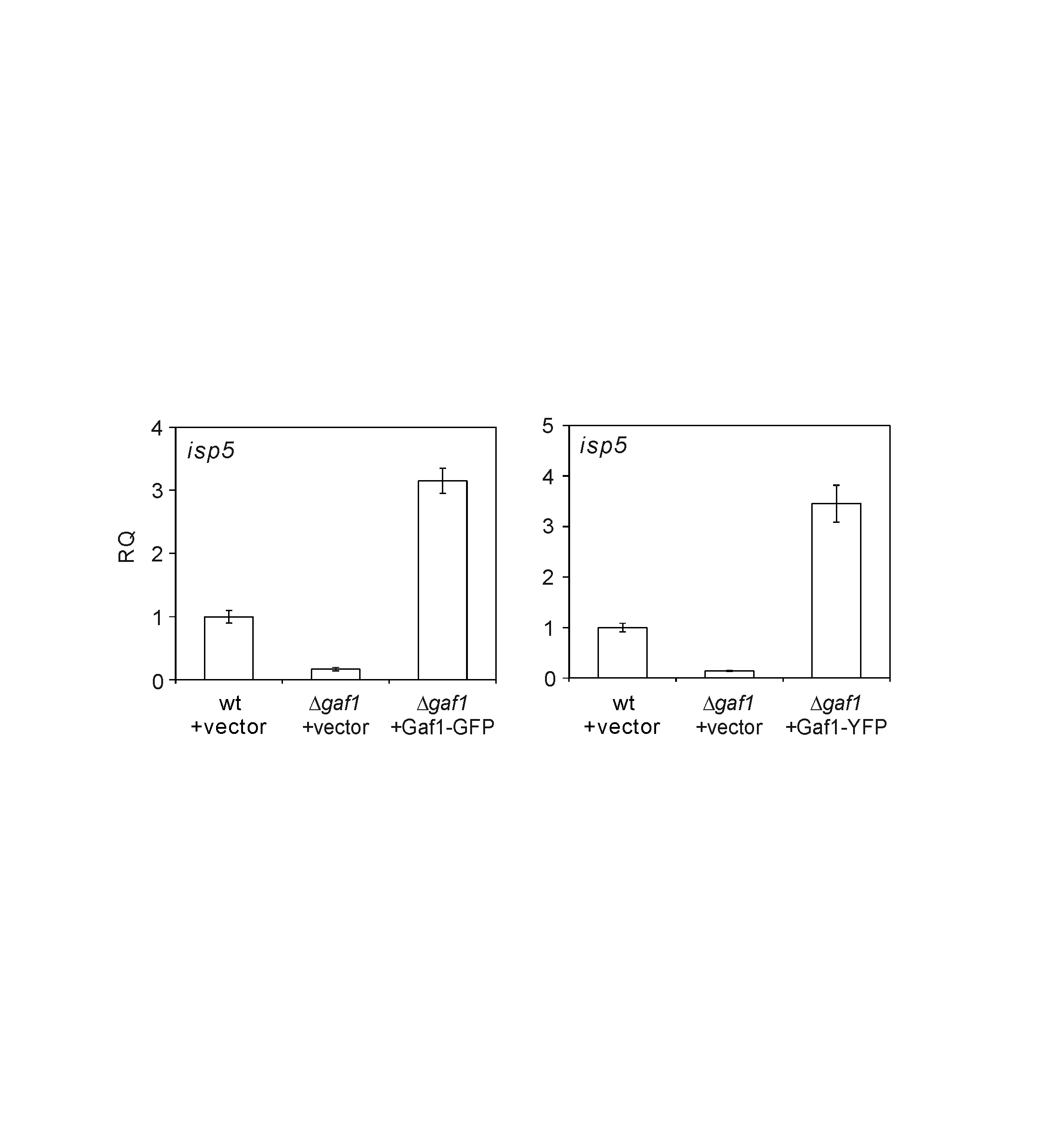

Supplement: S3 Fig — Wild-type (wt) cells harboring control vector, Δgaf1 cells harboring the control vector or the plasmids expressing Gaf1-GFP or Gaf1-YFP under the nmt promoter were grown overnight in EMM medium in the presence of 50 μM thiamine to early log phase, and then the cells were harvested. Total RNA was extracted and subjected to semi-quantitative RT-PCR for the indicated amino acid permeases. The values were obtained by the comparative CT method in comparison to those of act1, and then were normalized to those in wild-type cells (RQ: relative quantity). The values were averaged from three independent experiments and were shown. Note that weak, leaky expression of Gaf1-YFP or Gaf1-GFP in the presence of thiamine is sufficient to rescue defective isp5 + expression in Δgaf1 cells. (TIF) [file pone.0144677.s003.tif]

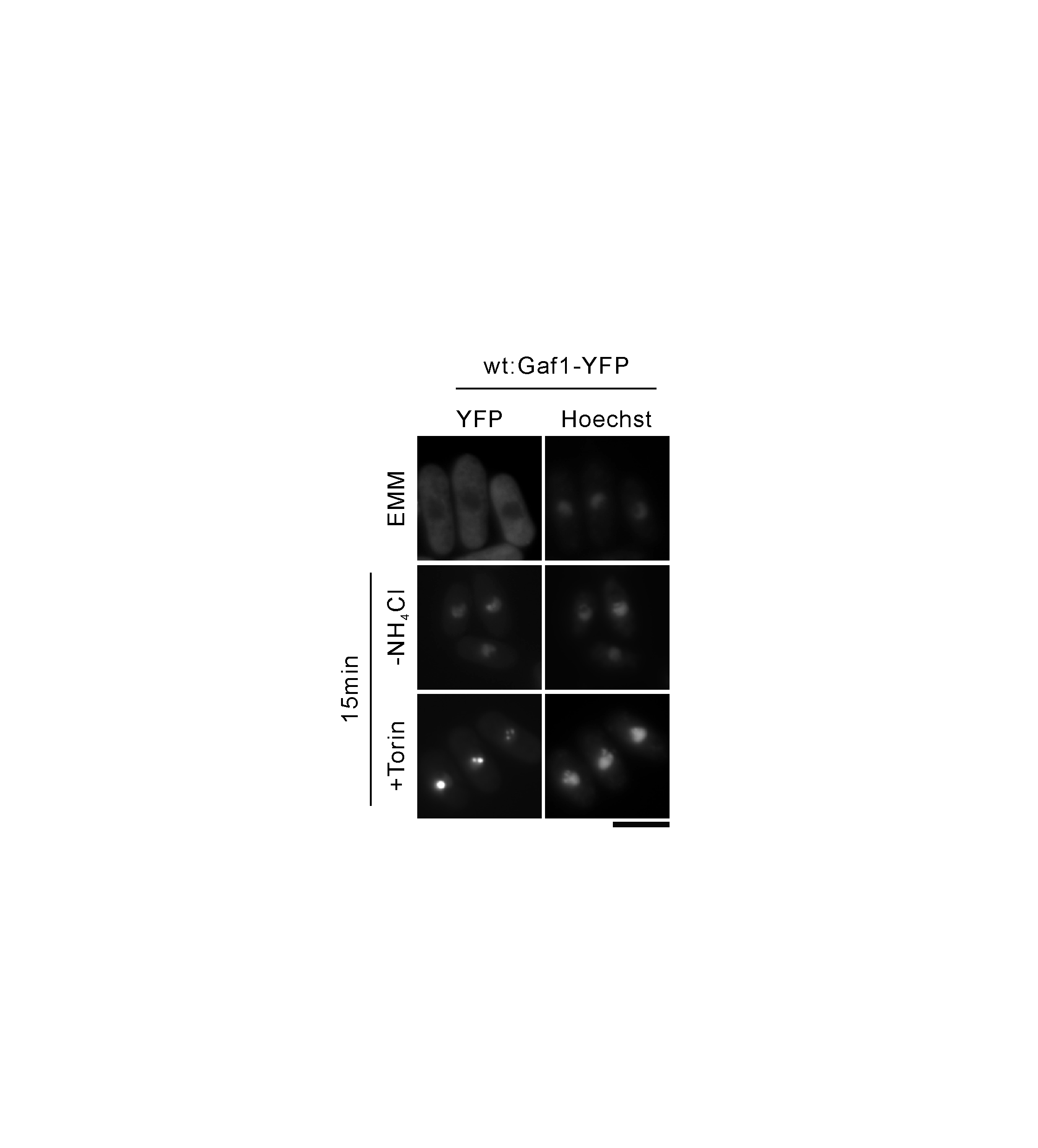

Supplement: S4 Fig — Wild-type (wt) cells expressing Gaf1-YFP under its native promoter were grown to early log phase in EMM medium at 27°C. The cells were treated without or with nitrogen depletion or Torin1 treatment (EMM, -NH4Cl and +Torin, respectively) for 15 min. Nuclear staining with Hoechst demonstrates nuclear localization of Gaf1-YFP induced by nitrogen depletion and Torin-1 treatment. Scale bar, 10 μm. (TIF) [file pone.0144677.s004.tif]
